# Exploring the influences of myopia in primary and secondary school students in Xinjiang using propensity score matching

**DOI:** 10.3389/fmed.2025.1537848

**Published:** 2025-05-14

**Authors:** Xiaopeng Hu, Xianyan Yuan, Hua Li, Haoxuan Gong, Zhicong Fu, Yuting Xie, Lin Zhu, Haina Chen, Yingli Yang, Dongsheng Rui

**Affiliations:** ^1^School of Medicine, Shihezi University, Shihezi, China; ^2^Shihezi City Centre for Disease Control and Prevention, Shihezi, China; ^3^Kindergarten No. 5, Zhangye, China

**Keywords:** myopia, primary and middle school students, risk factors, cross-sectional study, epidemoiology

## Abstract

**Objective:**

Due to the high prevalence of myopia among primary and secondary school students in Shihezi City in recent years, this study was conducted to understand the prevalence and the factors influencing it and to provide a scientific basis for future interventions to improve, protect, and promote the visual health of these students.

**Methods:**

The study population consisted of primary and secondary school students in Shihezi City. Stratified random sampling was employed for participant selection. Schools were first randomly chosen from both urban and rural areas of Shihezi City, followed by grade-level stratification within each selected school. Two classes per grade were randomly designated as sample classes. All students underwent vision screening. Students in Grade 4 and above completed questionnaires to investigate myopia-associated factors. To control for confounding effects, four variables**–**grade level, gender, urban/rural residence, and parental myopia status**–**were matched and analyzed using propensity score matching.

**Results:**

A total of 6,732 individuals were surveyed in this study, with 6,092 ultimately included (participation rate: 90.49%), of whom boys represented 51.10%. The overall prevalence of myopia was 59.55%. The prevalence of myopia in urban areas was 68.17%, while in rural areas, it was 42.42%, a difference that was statistically significant. Risk factors for myopia included being female, having myopic parents, engaging in close-distance reading or writing, sleeping less than 8 h per night, and receiving education at an older age. Protective factors against myopia included attending school in a rural area, watching television from a distance of more than 3 m, and having had a vision examination at least once in the past year.

**Conclusion:**

The myopia rates in Shihezi City exceed the national average and show a gradual increase with advancing school age. In addition to inherent factors such as gender, receiving education at an older age, and genetic predisposition, myopia prevention strategies should include proper sitting posture, good eye habits, and health promotion measures.

## Introduction

Myopia is recognized by the World Health Organization as one of the five major eye diseases that must be addressed or eliminated worldwide by 2020 (1), making it a major focus of global public health (2). Holden predicted that by 2050, myopia will affect 4,758 million people globally (approximately 49.8% of the total population) (3). In China, the prevention of myopia is an important public health task because the prevalence of myopia among Chinese school-age children is one of the highest in the world (4, 5). In 2020, the overall myopia rate among children and adolescents in China was 52.7%, reflecting an increase of 2.5% from 2019 (50.2%). Some studies predict that by 2050, the myopia rate among children and adolescents aged 3–19 years in China will reach 84.0% (6). This alarmingly high myopia prevalence compared to Western countries may be attributed to Chinese-specific culture, including parents’ educational philosophy, increasingly strict admission criteria, and children spending more time studying and less time outdoors (7). In addition to increasing the socio-economic burden, myopia not only affects the physical, psychological, cognitive, and social functioning and quality of life in China (5) but is also associated with the development of serious pathological diseases, such as macular degeneration, retinal detachment, glaucoma, and cataracts (8). The physical and psychological damage caused by vision problems in children and adolescents seriously affects the healthy development of human society.

In recent years, myopia has been reported among primary and secondary school students in some large cities in inland China (9–13). In contrast, myopia rates among primary and secondary school students in northwestern China, especially in Xinjiang, and the factors influencing it, have been rarely documented. Xinjiang has certain special characteristics compared to the mainland, with significant cultural and economic differences. As an important city in Xinjiang, Shihezi City offers a representative level of public health surveillance. In this context, we investigated the prevalence of myopia among elementary school children in Shihezi City, and we also considered the effects of gender and urban–rural distribution on myopia and explored the influencing factors of myopia to fill the gaps in existing knowledge about myopia in China. Our study provides valuable guidance and a theoretical framework for effective prevention and management of myopia in children and adolescents.

## Methods

### Subjects of the study

Data used in this project were obtained from the National Students’ Common Diseases and Health Influencing Factors Surveillance and Intervention in the Shihezi City Area, a project conducted by the Chinese Center for Disease Control and Prevention (CDC). The program was jointly administered by education and health departments at all levels, providing high-quality surveys and data. This study was conducted in 2022 and students from schools in Shihezi city were selected as the study population. A multi-stage stratified cluster sampling method was used. In the first step, the city was divided into urban and rural administrative units, and schools were selected from three categories: primary, junior high, and senior high schools. In the second step, stratified sampling was conducted according to grade, followed by class-level sampling to form the study sample. In the random cluster sampling, a certain number of classes were selected to meet the minimum requirements for the study and sample size. This included three elementary schools, five middle schools, and four high schools in urban areas, as well as three elementary schools and two middle schools in rural areas (where there are no high schools). All students underwent a vision examination, while only students in grades ≥4 of primary school completed the questionnaire. The final sample size comprised 6,092 students (3,113 boys and 2,979 girls), of whom 4,578 were in grades ≥4. Additionally, 4,511 and 2,039 students were from urban and rural areas, respectively. Each student’s parent or guardian was informed and provided written consent before the ophthalmological examinations. This study adhered to the tenets of the Declaration of Helsinki and was approved by the Ethical Review Committee of the First Affiliated Hospital of Shihezi University. Participants provided informed consent to participate in the study before taking part (KJ2024-036-02).

## Survey methods

### Visual acuity examination

A standard logarithmic visual acuity chart and computerized automated optometry TOPCON KR-1 (Tokyo, Japan) under non-ciliary muscle paralysis (non-dilated pupils) were used to test visual acuity in both the right and left eyes. The standard GB/T 11533–2011 logarithmic 5-point recording visual acuity chart was utilized. Refraction was assessed through an objective examination using desktop automatic computer optometry under non-ciliary muscle paralysis conditions. Three measurements were taken for each eye and averaged. The test results were recorded by the on-site testing physician in the CDC Student Common Diseases and Health Factors Monitoring Information System.

### Questionnaire

The questionnaire was developed by the Chinese CDC and completed on-site by students in grades ≥4. Before administering the questionnaire, the researcher explained its significance, emphasized the confidentiality of the responses, and asked students to maintain an appropriate distance from each other to ensure careful answering. The survey covered several aspects, including parental myopia, eye environment, eye habits, screen use, outdoor activities, and sleep (Schedule 2). The results of the questionnaire were directly uploaded to the CDC Student Common Diseases and Health Factors Monitoring Information System.

### Myopia screening criteria

According to the Chinese National Myopia Screening Standards, the diagnostic criteria for screening myopia included a standard logarithmic visual acuity of <5.0 in the naked eye (without correction) and a computerized optometric spherical equivalent visual acuity of <−0.50 D in the case of non-ciliary muscle paralysis (14). Individuals with myopia in at least one eye, or those wearing keratoconus lenses, were counted in the total number of myopia cases. Subjects wearing contact lenses were asked to remove them for the examination.

### Quality control

The screeners were certified national practitioners, technicians, or nurses specializing in optometry, all of whom had received relevant training. During the survey, retest subjects were randomly selected, constituting 5% of the students tested that day. Both non-corrected visual acuity and best-corrected visual acuity were reassessed.

### Data analysis

Data were double-entered into the database using the EpiData 3.1 parallel data entry method based on a standardized questionnaire provided by the CDC. The data were verified by the National Center for Disease Control and Prevention (NCDC). Statistical analysis was performed using SPSS 27.0. Count data were expressed as the number of cases (percentage), and analysis of variance for the count data was performed using the *χ*^2^ test. Myopia influences were analyzed using unconditional binary logistic regression, with myopia-related influences as the independent variable and the presence or absence of myopia as the dependent variable. Propensity score matching was based on matching baseline characteristics of students in the myopic and non-myopic groups. The determinants used to create the propensity scores included four variables: gender, grade level, urban/rural residence, and parental myopia. Unconditional binary logistic regression analyses were performed by matching students with the same propensity score on a one-to-one basis without replacement and equalizing the two groups by eliminating non-matched cases. The significance level was set at *α* = 0.05 (two-sided).

## Results

### Prevalence of myopia

#### Overall analysis

**Figure 1:**
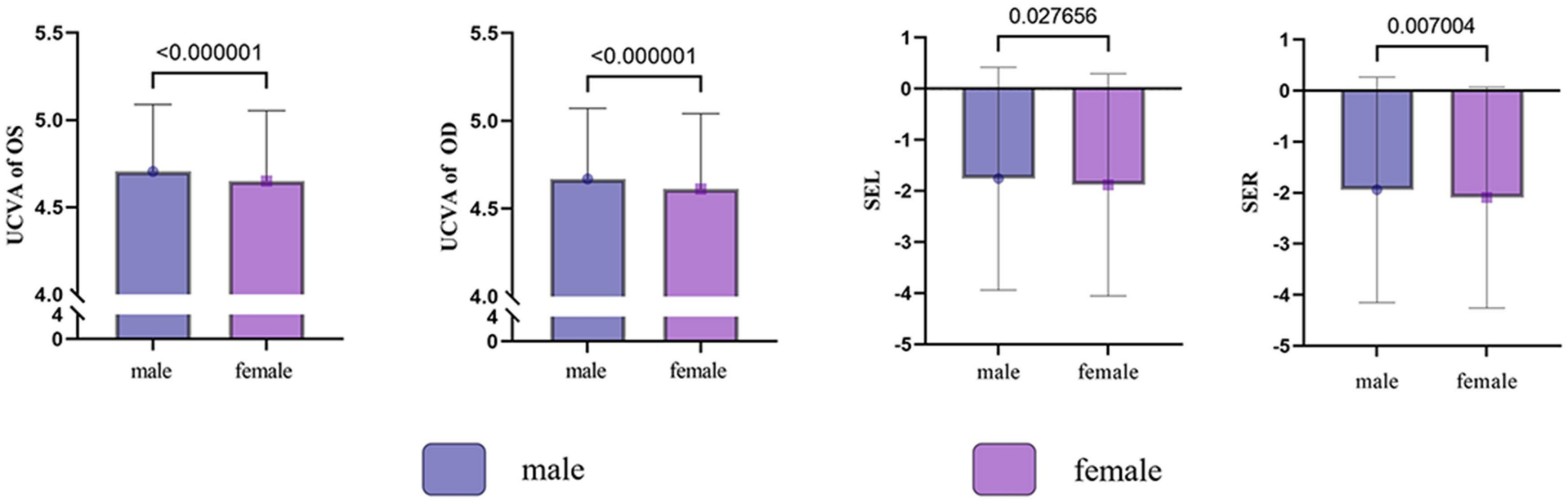
Gender differences in visual acuity and equivalent spherical lens power among primary and secondary school students in Shihezi.

A total of 6,092 students with a median age of 12.56 years (range: 9.65 to 15.07) were surveyed, with a male-to-female ratio of 51.10 and 48.90%, respectively. According to the screening results, 1,747 cases of myopia were detected in 3,113 male students, resulting in a myopia rate of 56.12% (1,747/3,113; 95% CI: 0.5436 to 0.5787). In female students, a total of 1,881 myopia cases were identified in 2,979 students, yielding a myopia rate of 63.14% (1,881/2,979; 95% CI: 0.6138 to 0.6488). The overall myopia rate was 59.55% (3,628/6,092; 95% CI: 0.5831 to 0.6079), with myopia rates of 41.01% in primary school, 74.11% in middle school, and 86.02% in high school. Additionally, the difference in myopia rates between male and female students was statistically significant (*χ*^2^ = 31.166, *p* = 0.001), as shown in Table 1 and Figure 1. The prevalence of myopia generally increased with the increase in grade.

**Table 1:** Current status of myopia among primary and secondary school students in Shihezi City in 2022.

Grade	Male		Female		Total		*χ* ^2^	*p*
	Total (N)	Myopia *n* (%)	Total (N)	Myopia *n* (%)	Total (N)	Myopia *n* (%)		
1	256	44 (17.19)	250	42 (16.80)	506	86 (17.00)	0.013	0.908
2	269	69 (25.65)	227	63 (27.75)	496	132 (26.61)	0.279	0.598
3	274	92 (33.58)	238	79 (33.19)	512	171 (33.40)	0.008	0.927
4	283	135 (47.70)	241	134 (55.60)	524	269 (51.34)	3.250	0.071
5	286	142 (49.65)	247	130 (52.63)	533	272 (51.03)	0.471	0.492
6	264	153 (57.95)	259	186 (71.81)	523	339 (64.82)	**11.013**	**0.001**
7	314	199 (63.38)	327	245 (74.92)	641	444 (69.27)	**10.034**	**0.002**
8	317	232 (73.19)	295	236 (80.00)	612	468 (76.47)	**3.943**	**0.047**
9	315	231 (73.33)	278	225 (80.94)	593	456 (76.90)	**4.804**	**0.028**
10	183	155 (84.70)	215	190 (88.37)	398	345 (86.68)	1.155	0.282
11	171	143 (83.63)	213	188 (88.26)	384	331 (86.20)	1.714	0.190
12	181	152 (83.98)	189	163 (86.24)	370	315 (85.14)	0.375	0.540
Total	3,113	1747 (56.12)	2,979	1881 (63.14)	6,092	3,628 (59.55)	**31.166**	**0.001**

1. Grades 1, 2, 3, 4, 5, 6, 7, 8, 9, 10, 11, and 12 correspond to primary school 1st grade, primary school 2nd grade, primary school 3rd grade, primary school 4th grade, primary school 5th grade, primary school 6th grade, middle school 1st grade, middle school 2nd grade, middle school 3rd grade, high school 1st grade, high school 2nd grade and high school 3rd grade, respectively. 2. The chi-square test was used to compare male and female students in the same grade. Bold indicates statistically significant.

**Figure 2:**
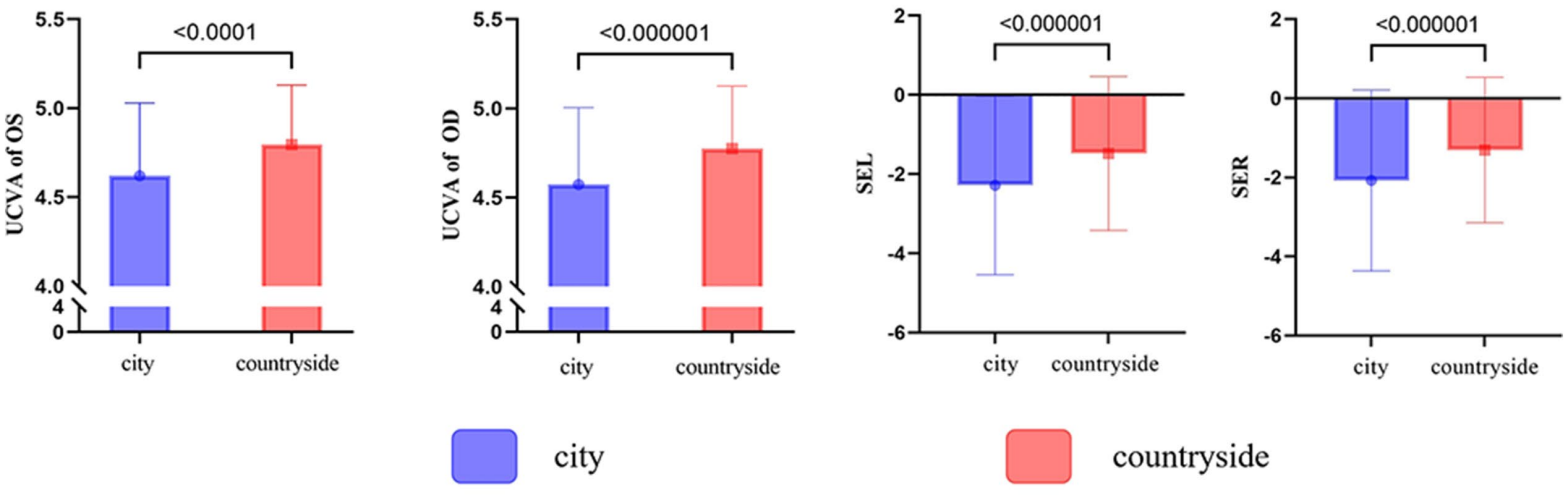
Urban/rural differences in visual acuity and equivalent spherical lens power among primary and secondary school students in Shihezi.

A total of 2,763 myopia cases were screened in 4,053 individuals in urban areas, resulting in a myopia rate of 68.17% (2,763/4,053; 95% CI: 0.6671 to 0.6960). In rural areas, 865 myopia cases were identified in 2,039 individuals, yielding a myopia rate of 42.42% (865/2,039; 95% CI: 0.4027 to 0.4460), as shown in Table 2 and Figure 2. Among the identified cases, the myopia rates between urban and rural areas for boys in grades 3–5 and junior grades, girls in grades 1–5, overall grades 1–5 and junior grades, and overall urban and rural populations were statistically different, as shown in Schedule 1.

**Table 2:** Urban–rural distribution of myopia among primary and secondary school students in Shihezi City, grouped by grade, 2022.

Grade	City		Countryside		*χ* ^2^	p
	Total (N)	Myopia *n* (%)	Total (N)	Myopia *n* (%)		
1	259	53 (20.46)	247	33 (13.36)	**4.522**	**0.033**
2	255	84 (32.94)	241	48 (19.92)	**10.761**	**0.001**
3	249	107 (42.97)	263	64 (24.33)	**19.973**	**0.000**
4	262	160 (61.07)	262	109 (41.6)	**19.869**	**0.000**
5	267	162 (60.67)	266	110 (41.35)	**19.905**	**0.000**
6	274	186 (67.88)	249	153 (61.45)	2.371	0.124
7	463	333 (71.92)	178	111 (62.36)	**5.523**	**0.019**
8	442	345 (78.05)	170	123 (72.35)	2.218	0.136
9	430	342 (79.53)	163	114 (69.94)	**6.127**	**0.013**
10	398	345 (86.68)	—	—	—	—
11	384	331 (86.2)	—	—	—	—
12	370	315 (85.14)	—	—	—	—
Total	4,053	2,763 (68.17)	2039	865 (42.42)	**373.392**	**0.000**

1. Grades 1, 2, 3, 4, 5, 6, 7, 8, 9, 10, 11, and 12 correspond to primary school 1st grade, primary school 2nd grade, primary school 3rd grade, primary school 4th grade, primary school 5th grade, primary school 6th grade, middle school 1st grade, middle school 2nd grade, middle school 3rd grade, high school 1st grade, high school 2nd grade and high school 3rd grade, respectively. 2. —: There’s no high school in the countryside. 3. The chi-square test was used to compare male and female students in the same grade. Bold indicates statistically significant.

### Exploration of factors influencing myopia

Only students in grade 4 or above completed the questionnaire: 1,514 participants were in grades 1–3, and 4,578 were in grade 4 or above. Univariate logistic regression analysis revealed that the following factors were associated with an increased risk of myopia: female sex, rural residence, higher school grade, greater height, higher body weight, infrequent seat changes, never adjusting seat height, spending >2 h on homework after school, reduced exercise time due to academic workload, parental non-restriction of electronic device use, poor writing posture, lack of teacher reminders for proper reading/writing postures, lack of parental reminders for postures, prolonged near-work without breaks, outdoor time < 1 h/day, parental myopia, no vision examination within the past year, reading/using electronic devices in sunlight, reading/using devices in dark conditions, reading/using devices while lying on one’s side, and reading/using devices while walking or riding. Conversely, sleep duration >10 h/day and outdoor activities during class breaks were protective factors against myopia. The details are presented in Table 3.

**Table 3:** Associations between myopia and various factors in primary and secondary school students in Shihezi.

Variables	Value	*p*	OR	95% CI for OR	
				Lower	Upper
Gender	1:Male 2:Female	**0.001**	**1.321**	**1.196**	**1.459**
District	1:City 2:Countryside	**0.001**	**0.333**	**0.299**	**0.371**
Segments	1:Primary schools 2:Middle school 3:High school	**0.001**	**2.901**	**2.695**	**3.123**
Height		**0.001**	**1.04**	**1.034**	**1.046**
Weight		**0.001**	**1.022**	**1.017**	**1.026**
Frequency of seat transfers	1:Weekly, fortnightly or monthly 2:Once a term or never	**0.011**	**1.367**	**1.076**	**1.737**
Adjusting tables and chairs to height	1: Once every 2–3 months, once a semester, or once a school year 2: Never	**0.001**	**1.55**	**1.37**	**1.754**
Location of recess activities	1: Inside the school building 2: Outdoors	**0.001**	**0.527**	**0.466**	**0.596**
Duration of homework after school	1: No homework, <1 h or 1–2 h 2: 2–3 h or > 3 h 3: Do not know (not involved in analysis)	**0.001**	**2.268**	**1.955**	**2.632**
Frequency of reducing exercise time due to learning	1:Never 2:Sometimes 3: Often	**0.003**	**1.19**	**1.06**	**1.335**
Do parents limit the time spent using electronic devices?	1:Yes 2:No	**0.001**	**1.612**	**1.397**	**1.859**
Reading and writing with the chest a punch away from the table	1: Always, often 2: Occasionally, never	**0.001**	**1.434**	**1.255**	**1.639**
Reading and writing with your eyes 33 cm away from the book	1: Always, often 2: Occasionally, never	**0.001**	**1.588**	**1.389**	**1.815**
Reading and writing with your fingers 3 cm from the tip of the pen	1: Always, often 2: Occasionally, never	**0.001**	**1.431**	**1.239**	**1.653**
Eyes more than 3 m away from the TV display when watching TV	1: Always, often 2: Occasionally, never	0.121	1.047	0.988	1.109
Does the teacher remind about reading and writing postures?	1: Always, often 2: Occasionally, never	**0.001**	**1.542**	**1.344**	**1.770**
Do parents remind about reading and writing postures?	1: Always, often 2: Occasionally, never	**0.004**	**1.229**	**1.069**	**1.414**
How often should you rest your eyes when loading your eyes up close	1: ≤15 min, >15 min but <0.5 h, 0.5–1 h 2: 1–2 h, 2–3 h, ≥3 h	**0.001**	**1.675**	**1.446**	**1.94**
Time spent outdoors during the day	1: ≥3 h, 2–3 h or 1–2 h 2: <1 h 3: Do not know (not involved in analysis)	**0.001**	**1.562**	**1.312**	**1.859**
Parental myopia	1: Neither parent is myopic 2: Only the mother or father is myopic 3: Both parents are myopic	**0.001**	**1.634**	**1.464**	**1.824**
How many vision tests have been done in the past year?	1: ≥1 2: 0	**0.005**	**1.312**	**1.088**	**1.583**
Reading books or electronic screens in direct sunlight	1: Always, often, or occasionally 2: Never	**0.001**	**1.538**	**1.348**	**1.755**
Turning off the lights when watching electronic screens after dark	1: Always, often, or occasionally 2: Never	**0.001**	**1.775**	**1.561**	**2.018**
Crouching or lying down to read a book or electronic screen	1: Always, often, or occasionally 2: Never	**0.001**	**1.524**	**1.347**	**1.724**
Reading books or electronic screens while walking or traveling in a car	1: Always, often, or occasionally 2: Never	**0.001**	**1.647**	**1.438**	**1.887**
Sleeping time	1: <8 h 2: 8–10 h 3: ≥10	**0.001**	**0.535**	**0.489**	**0.586**

Bold indicates statistically significant.

The univariate analysis results were evaluated using unconditional binary logistic regression. The multifactorial analysis showed that parental myopia and reading and writing with eyes no more than one foot away from the book were risk factors for myopia after removing the effects of gender, grade, and urban versus rural areas (Table 4).

**Table 4:** Factors associated with myopia in primary and secondary school students in Shihezi (multivariable analysis).

Variables	*β*	S.E.	Wald	*p*	OR	95% CI for OR	
						Lower	Upper
Constant	−0.506	0.941	0.289	0.591	0.603		
Gender	0.246	0.113	4.73	**0.03**	**1.278**	**1.025**	**1.595**
District	−0.543	0.154	12.442	**0.001**	**0.581**	**0.43**	**0.786**
Grade	0.825	0.116	50.707	**0.001**	**2.283**	**1.819**	**2.865**
Parental myopia	0.381	0.063	36.695	**0.001**	**1.463**	**1.294**	**1.655**
Reading and writing without keeping your eyes >33 cm away from the book	0.163	0.066	6.054	**0.014**	**1.177**	**1.034**	**1.341**

Bold indicates statistically significant.

### Factors influencing myopia after propensity score matching

Because the study population varied widely in terms of gender, urban/rural status, parental myopia, and baseline grade status, to eliminate the effects of covariates, we ended up conducting a multifactorial analysis of the 2,454 individuals after matching propensity scores for these four variables. The results of the multifactorial analysis are shown in Table 5. The results indicated that shorter sleep duration and reading distances of less than 33 cm were risk factors for myopia while receiving a visual acuity examination at least once in the past year and watching television from a distance of more than 3 m from the display were protective factors.

**Table 5:** Multivariate analysis of factors influencing myopia after matching.

Variables	*β*	S.E.	Wald	*p*	OR	95% CI for OR	
						Lower	Upper
Constant	0.326	0.436	0.561	0.454	1.386		
Reading and writing with your eyes 33 cm away from the book	0.298	0.151	3.903	**0.048**	**1.347**	**1.002**	**1.811**
Watching television at a distance of more than 3 m from the television display	−0.463	0.172	7.268	**0.007**	**0.630**	**0.450**	**0.881**
Received a vision examination ≥1 in the past year	−0.553	0.247	5.017	**0.025**	**0.575**	**0.355**	**0.933**
Sleeping time	0.267	0.099	7.343	**0.007**	**1.307**	**1.077**	**1.585**

Bold indicates statistically significant.

## Discussion

### The current state of myopia in Shihezi

In this study, the overall myopia prevalence was 60.66%, with 41.01% of primary school students, 74.11% of middle school students, and 86.02% of high school students affected—rates that exceed the national average. According to data released in 2020 by the National Health Commission of China, the overall myopia rate among Chinese children and adolescents was 52.7%, with 35.6% in primary school students, 71.1% in junior high school students, and 80.5% in senior high school students, reflecting an increase of 2.5 percentage points compared to 2019 (50.2%). Myopia incidence rates among Chinese children and adolescents not only rank among the highest globally but also continue to rise, with onset ages trending younger. Therefore, urgent measures are necessary to curb the rapid progression of myopia.

### Factors influencing myopia in primary and secondary school students in Shihezi are different

This study found that the prevalence of myopia tends to increase as the grade level advances. The highest myopia prevalence, 86.02%, was observed among high school students, likely due to the heavy school workload and prolonged, frequent periods of eye strain. This may also explain the difference in myopia prevalence between urban and rural students, as myopia prevalence is lower in rural students. This may be because rural students experience less study pressure compared to urban students.

In this study, a statistically significant difference in myopia prevalence was found between boys and girls (male: 57.43%, female: 64.06%, χ^2^ = 30.118, *p* = 0.001), with girls having a 1.445 times higher risk of developing myopia compared to boys, similar to the results of many studies (6, 15, 16). This gender difference may be due to boys spending more time outdoors than girls. However, it could also be influenced by social factors, as girls have had access to educational opportunities in recent years (17).

The present study showed a high genetic predisposition to myopia, consistent with previous studies demonstrating that children with myopic parents are more likely to develop myopia than those with non-myopic parents (18–20). Jones et al. (21) stated that myopia in one parent increases the risk of myopia in the child by a factor of 2.08, while myopia in both parents raises the risk by a factor of 5.07. However, the rapid increase in myopia incidence cannot be attributed to genes alone; environmental and behavioral factors are likely more significant contributors.

Compared to the past, young people are increasingly involved in indoor and sedentary activities, and electronic devices have an ever-growing presence in daily life. Additionally, more stringent entry barriers and the need for higher qualifications keep students in school for longer periods, leaving less time for relaxation.

We found that urban/rural status, gender, school age, and parental myopia had a significant impact on the results of the multifactorial analyses, affecting the inclusion of other variables in the model. Therefore, we attempted to eliminate the influence of these variables by using propensity score matching before conducting the multifactorial analysis. The results showed that keeping the eyes ≤33 cm away from the book while reading was a risk factor for myopia (OR = 1.347, *p* = 0.048). Furthermore, studies have demonstrated that a shorter reading distance leads to faster myopia progression (22), suggesting that maintaining good reading and writing postures and sleeping less than 8 h are beneficial in preventing myopia. These findings are consistent with previous studies (23, 24).

### Intervention efforts for myopia should consider population differences

Exploring the factors influencing myopia aims to improve future interventions and provide valuable guidelines for effective myopia prevention in children and adolescents. According to Lin et al. (10), myopia prevention in primary and secondary school students needs to be differentiated and targeted based on region, school type, and climatic conditions. Some inherent factors, such as family history, gender, and school age, cannot be changed. Additionally, it seems unlikely that the increasing demands for higher grades will allow children to reduce their workload. Therefore, our common aspiration is to prevent myopia without affecting students’ normal learning.

### How to prevent myopia

Based on our results, students need to sit upright and keep their hands 1 inch away from the tip of the pen, their chests 1 fist away from the table, and their eyes 1 foot away from the book while reading and writing. When engaging in prolonged eye activities, taking breaks, going outdoors for a few minutes, and looking into the distance are important. Recess activities should be outdoors as much as possible at school, as some studies show that outdoor activities can prevent myopia (25). It is also important to avoid putting strain on the eyes under poor lighting conditions, crouching or lying down to read, and reading while walking or traveling in a car. Teachers should encourage students to maintain good sitting posture in class or during studying and to go outdoors between classes, as some studies demonstrate that proper monitoring of outdoor activity adherence is essential for the protective effect of the intervention (26). Additionally, avoiding the reduction of time allocated to students’ physical education classes or outdoor activities is necessary. For parents, limiting the amount of time their children spend on electronic devices, such as mobile phones, tablets, and computers, is necessary. Furthermore, they should supervise their children at home to ensure they maintain good reading and writing postures and purchase eye-protecting desk lamps to ensure good lighting conditions when reading and writing.

### Strengths and limitations

This study was conducted as part of the National Monitoring and Intervention Program for Common Diseases and Health Influencing Factors of Chinese Students, ensuring methodological standardization and centralized quality control measures across all regions. The research team comprised uniformly trained professionals, further enhancing the reliability and validity of the findings. Methodologically, our use of propensity score matching to address confounding variables represents an innovative approach in this field. Notably, our inclusion of rural student populations distinguishes this study from previous studies that often overlooked this demographic. However, several limitations should be acknowledged. First, despite the substantial sample size, the cross-sectional design inherently restricts causal inference regarding myopia development. Second, as with many large-scale screening programs, ethical and logistical constraints precluded pupil dilation during refractive assessments, potentially leading to an overestimation of myopia prevalence. These limitations highlight the need for longitudinal studies and refined diagnostic protocols in future research.

## Conclusion

Myopia rates in Shihezi are higher than the national average and increase gradually with school age. In addition to inherent factors (gender, increasing school age, and hereditary factors), myopia prevention should include proper sitting posture, eye habits, and health awareness promotion. Furthermore, based on this study, myopia prevention measures should be tailored to different target groups.

## Data Availability

The raw data supporting the conclusions of this article will be made available by the authors, without undue reservation.
